# Emergent Multidien Cycles From Partial Circadian Synchrony

**DOI:** 10.1523/ENEURO.0464-25.2026

**Published:** 2026-07-15

**Authors:** Marc G. Leguia, Maxime O. Baud, Ralph G. Andrzejak

**Affiliations:** ^1^Life Sciences Department, Barcelona Supercomputing Center, Barcelona 08034, Spain; ^2^Department of Engineering, Universitat Pompeu Fabra, Carrer Roc Boronat 138, Barcelona, Catalonia 08018, Spain; ^3^Department of Neurology, Inselspital, University Hospital Bern, University of Bern, Bern 3010, Switzerland

**Keywords:** chronobiology, circadian rhythms, epilepsy, multidien cycle, network dynamics, neural oscillations

## Abstract

Over the past decades, chronobiology has attracted great attention thanks to the elucidation of the molecular mechanisms underpinning the circadian cycle. Now, growing evidence suggests that cycles longer than circadian, so-called “multidien” cycles, are of crucial importance in physiological fluctuations spanning multiple days with repercussions in health and disease. Unlike circadian clocks, multidien cycles may not be genetically encoded, given their heterogeneity within and across individuals and systems. Here, we propose that multidien cycles may be generated by the interaction between partially coupled circadian oscillators. To demonstrate this possibility theoretically, we use a ring model of coupled circadian oscillators and study how synchrony within this network evolves over time. We found that a free-running, about-weekly period robustly emerges from the network’s dynamics. A range of additional multidien cycles resulted from subtle variations in the coupling parameters within the network with periodicities reminiscent of those observed across different species. Thus, our model of emergent multidien cycles from partial circadian synchrony constitutes a credible hypothesis for explaining the timing of a myriad of events on the scale of weeks and months in health and disease.

## Significance Statement

Biological rhythms longer than a day, so-called “multidien” cycles, are increasingly recognized as critical to health and disease, yet their underlying mechanisms remain elusive. Our study offers a novel explanation: multidien rhythms may not require independent molecular clocks, but instead emerge naturally from partial synchrony among circadian oscillators. By linking chronobiology with non-linear dynamics, we demonstrate how complex, multi-day rhythms can arise through chimera states—patterns well-studied in physics but underexplored in biology. This mechanistic insight has the potential to transform the study of long-timescale biological phenomena, avoiding years of costly, reductionist research. Our findings provide a new theoretical foundation for multidien rhythms, relevant to researchers in neuroscience, physics, and systems biology.

## Introduction

Life is made of intricate biological cycles at multiple timescales. The field of chronobiology (Halberg, 1969), which studies the temporal structure of life, has made striking advances in elucidating the mechanisms (Bargiello et al., 1984; Zehring et al., 1984) of circadian clocks (Souêtre et al., 1989; Schulz and Steimer, 2009; McKenna et al., 2018) and their role in timing essential physiological functions in anticipation of day- and night-time. Recent evidence has shown that longer cycles co-exist with circadian rhythms, in humans (Ayers et al., 2014; Reinberg et al., 2017; Baud et al., 2018; Karaaslan et al., 2021; Karoly et al., 2021; Proix and Baud, 2021; Ceolini and Ghosh, 2023) and other animals (Schweiger et al., 1986; Bromage et al., 2016; Qualls et al., 2016; Baud et al., 2019; Gregg et al., 2020), yet their underpinnings have rarely been investigated (Labavić et al., 2017).

For example, about-monthly cycles may not be limited to women’s menstrual period, as similar timing also exists in testicular functions and hormones (Bittman, 2016). In addition, about-weekly cycles are found in tissue growth (Bromage et al., 2009; Karaaslan et al., 2021), cardiovascular functions (Watanabe et al., 2003; De Silva et al., 2026), electrolyte balance (Rakova et al., 2013) and hormonal secretion. The term “multidien cycles” was coined across fields of investigation (Bromage et al., 2016; Baud et al., 2018; Karaaslan et al., 2021; Proix and Baud, 2021) for quasi-rhythms spanning multiple days with variable but shared peak-periodicity centered around 7–10 d (“about-weekly” Reinberg et al., 2017) and/or 20–50 d (“about-monthly”) within and across individuals. In the absence of readily-identifiable external temporal cues (i.e., a “Zeitgeber”), these multidien cycles are likely generated endogenously, forming free-running oscillations in which the period-length varies from one cycle to the next. In general, multidien cycles are weaker than circadian cycles, but their importance has recently been emphasized in human health and disease (Yamashita et al., 1999; Karoly et al., 2021; Leguia et al., 2021; Proix and Baud, 2021).

Through the growing use of wearable and implantable monitoring technology, key insights were gained from physiological recordings in natural conditions over weeks, months, or years. Multidien cycles have now been characterized quantitatively in blood pressure (Reinberg et al., 2017), in the heart beat (smart-watch; Karoly et al., 2021), in the brain (chronic EEG; Leguia et al., 2021), as well as in human behavior, measured by smartphones (Ceolini and Ghosh, 2023). Highly relevant to medicine, multidien cycles can modulate heart rate by +/ − 20 beats per minutes (Karoly et al., 2021) with a potential impact on the timing of cardiovascular events (Yamashita et al., 1999; Zoghi et al., 2008). In the brain, multidien fluctuations in cortical excitability may underlie the periodic recurrence of seizures in epilepsy (Proix et al., 2021; Leguia et al., 2023; Xiong et al., 2023), headaches in migraine (Gonzalez-Martinez et al., 2024), and affective states in bipolar disorders (Wehr et al., 1988), to cite a few. Solid evidence is found in the field of clinical (Baud et al., 2018; Karoly et al., 2018; Maturana et al., 2020; Leguia et al., 2021) and experimental epilepsy (Baud et al., 2019; Gregg et al., 2021), where multidien cycles of epileptic brain discharges correlate so strongly with the occurrence of seizures that their monitoring enables the forecast of upcoming seizure risk (Maturana et al., 2020; Proix et al., 2021; Leguia et al., 2023).

Thus, multidien cycles are responsible for timing reproduction, vital physiological functions, and are of central importance in brain disorders. Yet, their underlying mechanism remains unknown. A yet-to-be-discovered independent multidien clock that synchronizes physiological systems over days seems unlikely. A rigid hierarchy would hardly explain the broad range of variable periodicities observed across organs. Instead, we here hypothesize that multidien cycles emerge from coupling relationships among many circadian clocks organized in a complex network (Labavić and Meyer-Ortmanns, 2017). We conjecture that the diversity of observed multidien rhythms could result from subtle modifications in coupling parameters, which we demonstrate using the Kuramoto model of coupled oscillators (Kuramoto, 1984; Kuramoto and Battogtokh, 2002; Andrzejak et al., 2017).

## Methods

### Model

**Figure 1.**
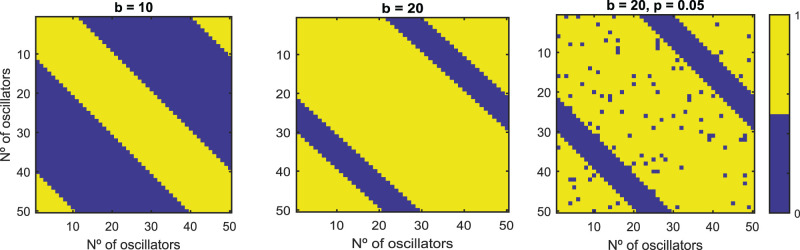
Network of coupled circadian oscillators. ***a***, Illustration of a *N* = 50 network with a coupling width of *b* = 10 and a coupling reduction of *p* = 0. ***b***, Same as ***a***, but with coupling width of *b* = 20. ***c***, Same as ***b*** but with coupling reduction of *p* = 0.05.

In our model, we simulate the interactions among *N* = 50 circadian oscillators (e.g., neurons, cells, and organs) that are partially coupled among them. We assume that the essential interaction between individual circadian oscillators can be described using only their phase. For this purpose, we use a known modification of the Kuramoto model (Kuramoto, 1984; Kuramoto and Battogtokh, 2002; Wolfrum and Omel’chenko, 2011; Andrzejak et al., 2017), in which the phase of each oscillator *ϕ*_*j*_(*t*) is described by:

$$\dot{\phi_{j}}(t)=\omega -\frac{1}{2b}\sum_{k=j-b}^{\;j+b}\sin\;(\phi_{\;j}(t)-\phi_{k}(t)+\alpha ),$$

where *ω* is the natural (here circadian) frequency of all coupled oscillators, *b* is the *coupling width*, i.e., how many neighbors on either side each oscillator is coupled to (Fig. 1), and *α* is the *phase lag* parameter. To assess how dynamics may be affected by disrupting the network, we introduce a *coupling reduction* factor *p*, the probability that any link will be removed. An illustration of the network parametrization by *b* and *p* is shown in Figure 1.

For additional analyses, we add a Zeitgeber (i.e., an external pacemaker) to all oscillators in Equation 1:

$$\dot{\phi_{j}}(t)=\omega -\epsilon \sin\;(\phi_{\;j}(t)-\psi (t)+\alpha_{D}(t))-\frac{1}{2b}\sum_{k=j-b}^{\;j+b}\sin\;(\phi_{\;j}(t)-\phi_{k}(t)+\alpha ),$$

where ϵ is the coupling with the external temporal cue *ψ*(*t*), a Zeitgeber with the same natural frequency as the oscillators to mimic synchronization of the circadian rhythm by daylight. Unless stated otherwise, all oscillators are set to the free-running circadian frequency *ω* = 2*π*/24 in the absence of a Zeitgeber ( ϵ=0), with the time-dependent phase lag *α*_*D*_(*t*) also held at zero. All simulations of the model are done using a fourth-order Runge–Kutta method with an integration step size of *dt* = 0.05 (72 min) and a length of simulations *t* = 5,000 d. The initial conditions are drawn from a random uniform distribution between 0 and 2*π*. The overall synchronization of all oscillators is measured by the Kuramoto order parameter (Kuramoto, 1984), also referred to as the synchronization index (Tass et al., 1998), phase-coherence (Hoke et al., 1989), or phase-locking value (Lachaux et al., 1999) in the literature:

$$R(t)e^{i\Phi (t)}=\frac{1}{N}\sum_{i=1}^{N}e^{i\phi_{i}(t)},$$

where Φ(*t*) is the average phase and *R*(*t*) is the magnitude of the Kuramoto order parameter, which can take values from *R*(*t*) → 0 for a completely asynchronous to *R*(*t*) = 1 for a completely synchronous system of oscillators. As we want to evaluate how *R*(*t*) varies over time, we are only interested in configurations that do not lead to a synchronous state [0 < *R*(*t*) < 1] and exclude the realizations that collapse to the fully synchronous state [*R*(*t*) = 1] within the simulation time.

Additionally, we model a point-process in which each oscillator “spikes” or “releases” at *ϕ*_*j*_ = 0 to mimic discrete communication between the elements of the system. The computation of the oscillators’ instantaneous overall spiking rate adds interpretability to the overall synchronization *R*(*t*). Thus, spiking rate *I*(*t*) is computed as the mean of oscillators that crossed the *ϕ* = 0 threshold at each time step of the simulation.

Finally, we introduced variability in the intrinsic frequency of each oscillator by perturbing the baseline natural frequency with multiplicative Gaussian noise. In practice, for a baseline frequency *ω*, we defined the oscillator-specific frequencies as:

$$\omega_{i}=\omega \left(1+\sigma \xi_{i}\right),\;\xi_{i}∼N(0,1),$$

where *σ* controls the amount of heterogeneity, so the frequencies have a normal distribution centered at 1 with standard deviation *σ*, resulting in unequal natural frequencies for each oscillator centered around the reference value *ω*. Unless stated otherwise, we keep the variability at zero.

### Time-frequency analysis

In our simulation setup, we are interested in how the magnitude of the Kuramoto order parameter *R*(*t*) varies over time and whether this variation has some periodicity. To resolve the signal’s instantaneous phase [angle of the Kuramoto order parameter, *e*^*i*Φ(*t*)^] and power [absolute value of the Kuramoto order parameter, *R*(*t*)] at each timepoint on a multidien timescale, we computed a Morlet wavelet transform on *R*(*t*) [or *I*(*t*)] between 4 and 70 d and its corresponding periodogram averaged over the simulation time. The first 20,000 integration steps (in units of *dt*, corresponding to 1,000 simulated days) are discarded to avoid initialization transients.

### Surrogate testing

Our null hypothesis *H*_0_ is that the observed periodicity in *R*(*t*) arises from random fluctuations and is not a true feature of the system. To test this, we construct surrogate timeseries by randomly shuffling the original real valued signal *R*(*t*) in time. The surrogate signal *R*(*t*)_surro_ retains the original amplitude but has lost all the temporal correlations that may have generated the observed periodicity. We then use the wavelet transform on the surrogate signal *R*(*t*)_surro_ and compare the periodograms of the surrogate signal to the periodogram of the original signal *R*(*t*). We generate 200 such surrogates, and statistical significance is considered for peaks in the true periodogram that are above the 95th percentile power in the surrogate periodograms.

### Clustering

Since the system we study has several parameters involved and each set of parameter configurations may lead to different periodicity of both *R*(*t*) and *I*(*t*), we use clustering of the periodograms to extract common patterns across different sets of parameters. Similar to methods in Leguia et al. (2021), we used non-negative matrix factorization (NNMF; Sra and Dhillon, 2005) as an unsupervised clustering algorithm to extract spectral patterns across parameter configurations. Each configuration of parameters *u* is represented by *Q*_*us*_ with *s* corresponding to the indices of the periodogram of length *S* of the Kuramoto order parameter *R*(*t*) calculated with the wavelet transform. We concatenate each configuration periodogram *Q*_*u*_ such as:

$$M=[Q_{1}Q_{2}\ldots Q_{u}\ldots Q_{L}],$$

where *L* is the total number of possible configurations of parameters and *M* has *L* × *S* size. Since matrix *M* does not have negative values, we approximate it (“factorize”) by two lower-dimensional matrices:

M≈WH,

where *W*_*S*_ × *k* and *H*_*k*_ × *L* are the factorized matrices obtained by iterations minimizing the root mean square residual to the original matrix *M*. The matrix *W*_*S*_ × *k* contains the *k* centroids corresponding to the most representative periodograms of length *S*. The corresponding *k* coefficients to approximate each periodogram in *M* are in matrix *H*_*k*_ × *L*. In our configuration, we use *k* = 5 different clusters to compare them with the clusters found in our previous work (Leguia et al., 2021).

### Code accessibility

All scripts used to reproduce the results presented in this manuscript are publicly available at https://github.com/MGrauLeguia/Emerging_multidien_cycles.

## Results

**Figure 2.**
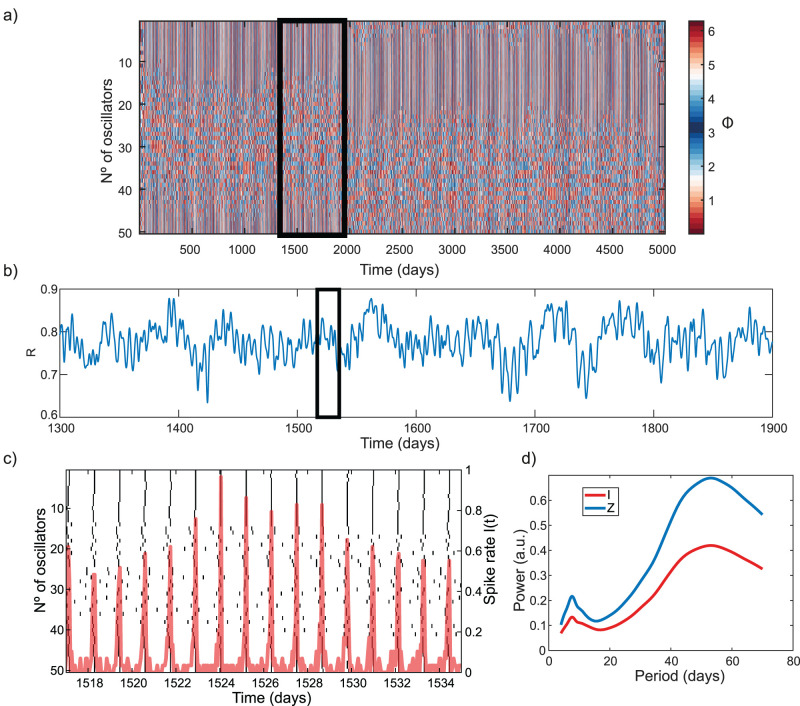
Coupled circadian oscillators generate multidien cycles. ***a***, Partial synchronization formation (so-called “Chimera state”) in the interaction of *N* = 50 oscillators with coupling width *b* = 18 and phase lag *α* = 1.46. ***b***, Kuramoto order parameter oscillations over the window depicted in ***a***. ***c***, Discretization of the spiking rates *I*(*t*) across all the oscillators (spiking at *ϕ* = 0) over the window depicted in ***b***. The trace in red shows fluctuations in the spiking rate (*y*-axis on the right) at circadian (within each day) and multidien (across days) timescales. ***d***, Periodicity of the Kuramoto order parameter *R*(*t*) and the spiking rate *I*(*t*) over the simulation period.

To obtain a system of *N* = 50 coupled oscillators that are partially synchronized (Abrams and Strogatz, 2004; Andrzejak et al., 2017), we simulate the model in Equation 1 for coupling widths *b* ∈ [14, 22] oscillators and a phase lag *α* ∈ [1.4, 1.60] radians, mimicking a network in which each element exchange delayed phasic signals (by 9̃0°) with its neighbors (30–40% of the network). For example, in the case of the system of oscillators with *b* = 18 and *α* = 1.46, we observe a dynamic and partial synchronization behavior with some oscillators synchronized and others de-synchronized at any timepoint (Fig. 2*a*)—a so-called chimera state (Abrams and Strogatz, 2004; Andrzejak et al., 2017). In this state, the Kuramoto order parameter oscillates around *R*(*t*) ≈ 0.8 (Fig. 2*b*) without entering full synchrony. We also simulate a point-process *I*(*t*) in which each oscillator “spikes” at a given phase, resulting in similar collective dynamics (Fig. 2*c*). Importantly, fluctuations in the overall synchronization (Fig. 2*b*) and spiking rates (Fig. 2*c*) showed emergent periodicity centered at ∼7 and ∼45 d for this set of parameters (Fig. 2*d*).

Specifically, the spiking rate *I*(*t*) yields a fast oscillation (circadian) modulated by a slower one (multidien) which resembles periodic fluctuations in inter-ictal epileptiform activity recorded with chronic EEG devices analyzed by us (Baud et al., 2018; Leguia et al., 2021) and others (Maturana et al., 2020; Karoly et al., 2021) in patients with focal epilepsy. Intuitively, firing neurons that lack complete circadian synchronization self-generate slower modulation of their concerted firing. This simulation result indicates that a system of coupled circadian oscillators, each spontaneously oscillating with identical periodicity but at a different phase, is sufficient to generate multidien cycles. As the order parameter *R*(*t*) and spikes *I*(*t*) share the same periodicities, subsequent analyses focused on *R*(*t*).

**Figure 3.**
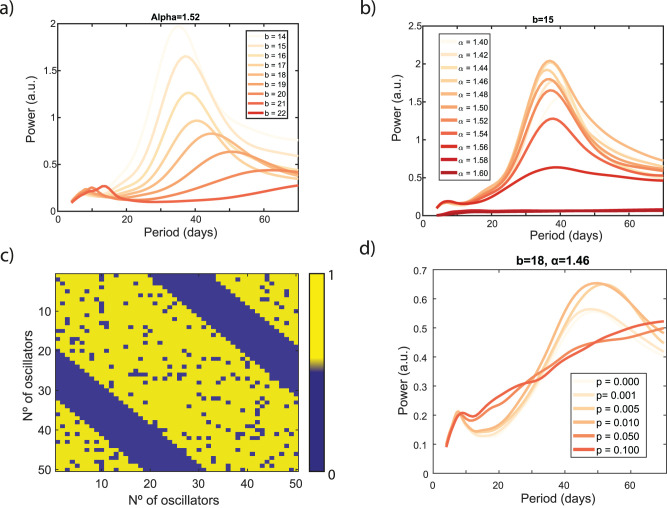
Modulation of multidien cycles by varying circadian coupling. The coupling width *b*, the phase lag *α*, and coupling reduction *p* contribute differently to the period and power in *R*(*t*). ***a***, Periodogram of the Kuramoto order parameter *R*(*t*) as a function of the coupling width *b* keeping the phase lag parameter fixed at *α* = 1.52. ***b***, Same as ***a*** but fixing *b* = 15 and changing the phase lag parameter *α*. ***c***, Example of the connectivity matrix with randomly removed links for a coupling reduction probability of *p* = 0.1. ***d***, Same as ***a*** with *α* = 1.46 and *b* = 18 with different coupling reduction probabilities *p*. The analysis on the different natural frequencies and their heterogeneity can be found in the Extended data Figures 3-1 and 3-2, respectively. The analysis on the sizes of the network is found in the Extended data Figure 3-3.

To characterize periodic fluctuations in the Kuramoto order parameter *R*(*t*) for a range of parameters, we average 10 simulations of the system in Equation 1 for pairs of coupling widths *b* and phase lags *α*. By fixing *α* = 1.52 radians, and varying the coupling width *b*, we observe systematic changes in the periodicity of *R*(*t*). In particular, the broader the coupling width, the longer the multidien periods (Fig. 3*a*). This suggests that basic network characteristics, such as connection density may underpin the range of observed multidien periodicities across systems (Baud et al., 2018; Maturana et al., 2020; Karaaslan et al., 2021; Karoly et al., 2021; Proix and Baud, 2021; Ceolini and Ghosh, 2023). Inversely, by fixing the coupling width at *b* = 15 oscillators and varying the phase lag, we observe varying multidien cycle strength in *R*(*t*) (Fig. 3*b*) with phase lags close to *π*/2 (anti-phase) intuitively abating multidien periodicity, as partial synchronization is known to disappear for *α* > *π*/2 (Abrams and Strogatz, 2004; Kotwal et al., 2017). This indicates that specific delays in biological communication between different circadian clocks (e.g., synaptic transmisison, endocrine secretion) may influence the strength of multidien cycles. Multidien periodicities also arise when all oscillators share a natural frequency tested over the range of 20–28 h (Fig. 3-1), showing that the effect found is not restricted to exactly 24 h, but valid for circa- (about) dian (24 h) periods found in biology. In contrast, heterogeneity in the natural frequencies among coupled oscillators disrupts multidien periodicity, increasingly so for wider frequency dispersion (Fig. 3-2). This suggests that heterogeneity in the natural frequencies can support multidien cycles over a finite range, but when it becomes sufficiently large, it disrupts the collective dynamics and destroys the quasi-periodic pattern. In general, the maintenance of partial synchronization also depends on consistent symmetric coupling (Yao et al., 2013; Ruzzene et al., 2019). To quantify this, we randomly removed links within the network and evaluated the effect on the Kuramoto order parameter *R*(*t*) (Fig. 3*c*). For a fixed coupling width *b* and phase lags *α*, the periodogram is stable for a low coupling reduction *p*. It loses the longer periodicity (45 d in the example) when the coupling reduction is *p* = 0.05 (Fig. 3*d*). These effects were consistently found across networks of different sizes (N ∈ [20,500]) (Fig. 3-3).

10.1523/ENEURO.0464-25.2026.f3-1 Figure 3-1 **Changing the natural frequency around the 24 h period also generates multidien periodicities** a) Periodogram of the model with driving using ɛ = 0.05, *b* = 15 and α = 1.42 as a function of the natural frequency ω_0_. The green line depicts our original setup with the natural frequency equal to the driver frequency. Solid (dashed) depicts periods lower (higher) than the natural frequency. Download Figure 3-1, TIF file.

10.1523/ENEURO.0464-25.2026.f3-2 Figure 3-2 **Strong heterogeneity destroys the emergence of multidien rhythms** a) Time evolution of the order parameter for different levels of natural-frequency heterogeneity. b) Periodogram of the model with different levels of noise strength σusing *b* = 15 and α = 1.48 Download Figure 3-2, TIF file.

10.1523/ENEURO.0464-25.2026.f3-3 Figure 3-3 **Size of the network does not affect the multidien rhythms** Periodogram of the order parameter *R*(*t*) for network sizes *N* ∈ {20, 50, 100, 200, 500}. Line color progresses from light to dark red with increasing *N*, with coupling parameter *b* = 0.3*N* (scaled such that *b* = 15 at *N* = 50) and α = 1.48 fixed throughout. A dominant peak around 35 days is consistently recovered across all system sizes, confirming the robustness of the oscillatory dynamics. The modest broadening observed for *N* = 20 reflects reduced network stability at small sizes. Download Figure 3-3, TIF file.

**Figure 4.**
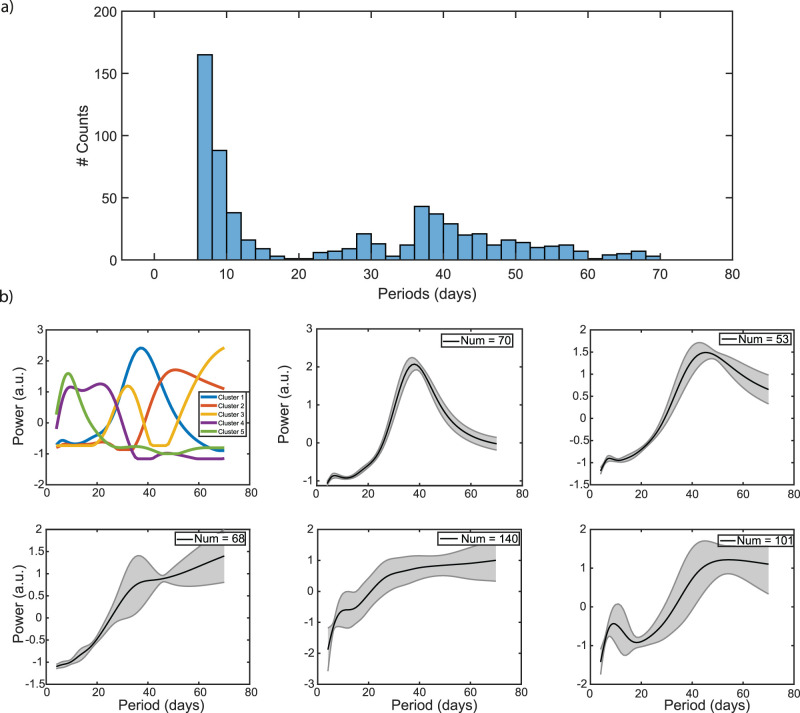
Multidien periodicities found across the parameter space. ***a***, Histogram for all significant peaks (assessed by testing against 200 surrogate timeseries, methods) calculated for all combinations varying *α*, *b*, and *p*. ***b***, Five clusters were extracted using NNMF. The average periodograms for all the combinations of variables in each of the five clusters are also shown in black with their standard deviation as the gray area.

In the parameter space of our model and for all different configurations of the system changing *α*, *b*, and *p*, we systematically observed the emergence of about-weekly and about-monthly peak-periodicity in agreement with Halberg’s free-running circaseptan rhythm (Schweiger et al., 1986; Ayers et al., 2014; Reinberg et al., 2017; Cornelissen, 2021), historical circalunar rhythms (Raible et al., 2017) and other evidence (Wehr et al., 1988; Bromage et al., 2016; Karaaslan et al., 2021; Karoly et al., 2021; Leguia et al., 2021; Fig. 4*a*). Furthermore, using the NNMF unsupervised clustering algorithm, we extract five component periodograms across all parameters studied and average periodograms belonging to the same cluster (Fig. 4*b*). While comparing real data directly to a parameter space exploration must be done with caution, the average periodicities intriguingly resembled those observed in real brain recordings (Leguia et al., 2021; Fig. 4*b*), mixing about-weekly and about-monthly periodicities in different proportions. Thus, we find that interactions of circadian oscillators can produce known patterns with attractor periodicities of about a week and about a month.

**Figure 5.**
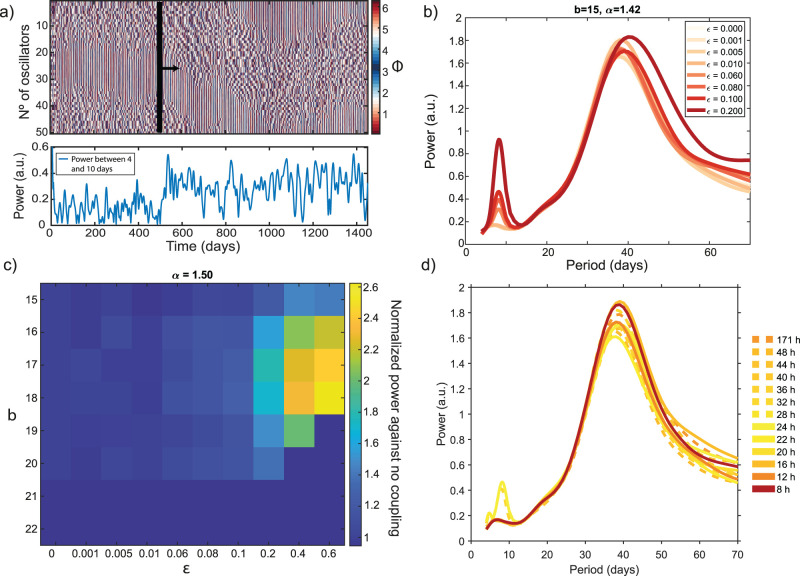
The addition of a circadian Zeitgeber in the system of oscillators enhances the magnitude of multidien cycles. ***a***, Partial synchronization example with an arrow showing when the Zeitgeber is added to the system of oscillators ( ϵ=0.1). The lower plot depicts the overall power between 4 and 10 d of the order parameter oscillations. ***b***, Periodogram of the order parameter as a function of Zeitgeber strength ϵ, keeping *b* = 15, and *α* = 1.42. ***c***, Change of the power in the multidien range keeping *α* = 1.50 and varying the coupling width *b* and the Zeitgeber strength ϵ. ***d***, Periodogram of the order parameter as a function of the driver period, keeping ϵ=0.1, *α* = 1.42, and *b* = 15. Thick lines correspond to Zeitgeber-period longer than 24 h and dotted lines to Zeitgeber-period shorter than 24 h, with redder colors the bigger the separation from 24 h. The analysis on the importance of the phase of the driver is found in the Extended data Figure 5-1.

So far, we simulated the system of Equation 1 in the absence of a Zeitgeber, i.e., keeping ϵ=0 to understand free-running periodicity. To characterize the impact of a Zeitgeber on multidien cycles, we now introduce pacing to all oscillators as in Equation 2 at *t* = 500 d, while keeping other parameters fixed. Immediately upon introduction of a circadian Zeitgeber, multidien cycles become stronger Figure 5*a* and the spiking *I*(*t*) synchronize to the Zeitgeber (Fig. 5-1). In particular, we observe that the stronger the Zeitgeber, the stronger the about-weekly cycle, while leaving the about-monthly cycle unaffected (lower plot in Fig. 5*b*) for different combinations of parameters *b* and *α* (Fig. 5*c*). To assess the importance of frequency-matched pacing, we varied the Zeitgeber’s period and examined its effect on the magnitude of emergent periodicities. We observed that about-weekly periodicity is strengthened only when the Zeitgeber frequency is close to the natural frequency of the system (here 24 h). Even a 7-day (i.e., calendar week) did not strengthen the magnitude of endogenously generated multidien cycles. This shows that a Zeitgeber specifically set at around 24 h can enhance self-organized multidien cycles of about a week, while leaving about-monthly periodicity unaffected.

10.1523/ENEURO.0464-25.2026.f5-1 Figure 5-1 **The IEA locking with the sun has a similar effect as the change of locking of *I*(*t*) with the time as** α*_D_*(*t*) changes. a) Locking between the simulated IEA and the time of the day while changing α*_D_* between 0 and π/6 in one year of simulation. Each arrow represents a monthly average locking, which corresponds to 600 data points. b) Locking between the IEA and the time of the day for different ǫ keeping α*_D_* = −π/6. Download Figure 5-1, TIF file.

**Figure 6.**
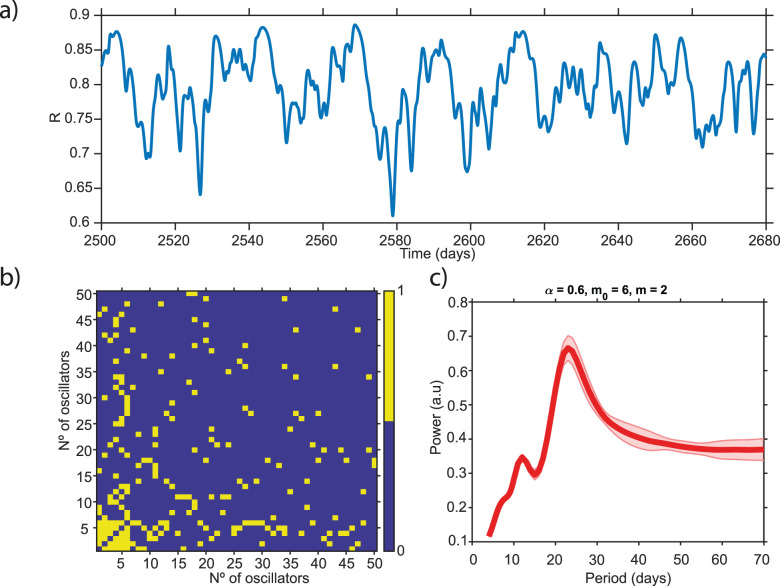
Multidien cycles are found in scale-free networks. ***a***, Order parameter across time of a window of the simulation. ***b***, Barabási–Albert network example for *m*_0_ = 6, and *m* = 2. ***c***, Periodogram of the order parameter for the system simulated with the network in ***b*** and with *α* = 0.6.

Throughout the manuscript, we explore ring network topologies, as they represent the canonical setting in which partial synchronization and resulting chimera states have been studied most extensively. However, biological systems such as connected brain oscillators (He Biyu, 2014) or networks of protein-protein interactions (Wuchty, 2001) often exhibit scale-free topology, characterized by a few highly connected hubs and many weakly connected nodes. To account for this, we simulated a network generated via the Barabási–Albert preferential attachment algorithm (Barabási and Albert, 1999; Fig. 6*b*): starting from *m*_0_ initial nodes, at each step a new node is added and connects to *m* existing nodes with a probability proportional to their degree—the so-called “rich-get-richer” mechanism that gives rise to the scale-free degree distribution. Although a full exploration of the parameter space would require a more extensive study, we found a parameter set for which the system does not reach full synchronization [0 < *R*(*t*) < 1], as shown in Figure 6*a,b*. Under these conditions, significant multidien periodicities (Fig. 6*c*) in *R*(*t*) emerge, demonstrating that multidien cycles are not exclusive to ring topologies.

## Discussion

Here, we showed how multidien cycles may emerge from the interactions among a network of partially coupled circadian oscillators. We propose that the network’s variable connection density, and signaling lags suffice to give rise to a range of multidien periodicities reminiscent of those observed across health (Levi and Halberg, 1982; Schweiger et al., 1986; Reinberg et al., 2017) and disease (Baud et al., 2018; Maturana et al., 2020; Karoly et al., 2021) in different species (Baud et al., 2019; Gregg et al., 2021; Leguia et al., 2021). This model of emerging, self-organized multidien cycles represents a credible alternative to that of a separate master multidien clock that would entrain physiological systems throughout the body. Indeed, it reconciles with the intuition that variable multidien cycles may not be genetically encoded. Furthermore, partial synchrony can be found in circadian gene expression across tissues, where phases cluster around a common mean while a minority of tissues display divergent phases (Talamanca et al., 2023).

As a complex network of many circadian oscillators enter into a state in which some oscillators are synchronized whereas others are not, momentary dynamic interactions generate fluctuations whose period-length varies from cycle to cycle. Resulting from this “chimera state,” multidien cycles are quasi-rhythms that are non-stationary but revolve around a central period, set by the individual tuning of network parameters. The resulting dynamics can fully account for the variability in multidien periods observed in real data within and across individuals, without the need to invoke genetic factors, lifestyle, the environment, or drug intake.

Remarkably, an about-weekly period (circaseptan) was the multidien period found most consistently across the parameter space tested, even in the absence of an environmental Zeitgeber. Many earlier studies sought explanations for about-weekly rhythms in the rigid organization of the social week (Ayers et al., 2014). Reversing the cause-effect relationship, our model rather suggests that physiological about-weekly rhythms may be endogenously generated and strengthened by the 24 h light–dark environment, which may have led humans to adopt a calendar week of seven days. Second in order of prevalence, about-monthly cycles were the strongest in magnitude, as found in data. Our observation of their endogenous emergence helps settle the long-standing debate about lunar influences on physiology in terrestrial animals (Zimecki, 2006). Thus, a prediction testable in experiments is the expected strengthening of certain multidien cycles, as a subject is moved from a constant to a cycling environment.

Limitations of this study relate to the assumptions made in our simplified canonical model to address essential chimera dynamics among a relatively small-sized network with a ring topology (neighbors are connected), an idealization of biological network architecture. In reality, myriads of cellular clocks do not necessarily have the same periodicity as Zeitgebers and may respect a hierarchy of coupling strengths, which may be the focus of future, more complex models. Exploratory simulations on larger and/or scale-free Barabási–Albert networks of circadian oscillators similarly revealed the emergence of multidien rhythms, giving credence to the generalizability of our findings. Our foundational study leaves many open questions: could inter-individual variability in multidien cycles result from such subtle parameter changes, with important consequences for rhythmicity in the system as a whole? Even within an individual, could different systems or organs have different multidien cycles, due to such differences? Could multidien cycles change over time due to illness, age, or environmental factors?

In conclusion, our proposed model represents a mechanistic framework to address the complexities of increasingly observed multidien cycles in health and disease. In the Era of long-term physiological monitoring, it is crucial that hypotheses are well formulated and costly experimental interventions at multidien timescale are soundly motivated. The successful management of chronic dynamical disorders in the fields of cardiology, psychiatry, or neurology may depend on the in-depth understanding of complex dynamics arising from biological rhythms.

## Synthesis

Reviewing Editor: Catherine Schevon, Columbia University Decisions are customarily a result of the Reviewing Editor and the peer reviewers coming together and discussing their recommendations until a consensus is reached. When revisions are invited, a fact-based synthesis statement explaining their decision and outlining what is needed to prepare a revision will be listed below. The following reviewer(s) agreed to reveal their identity: NONE.

Two reviewers evaluated the manuscript "Emergent multidien cycles from partial circadian synchrony." The authors describe a theory for how long cycles, with periods extending from weeks to months, could emerge from a circadian rhythm.   Both reviewers agreed that the manuscript is thought-provoking and provides a compelling account for a clinically relevant issue. The reviewers raised a few issues which are detailed below. Addressing these issues should strengthen the rigor of the manuscript and clarify the analyses and interpretations of the work.

Major Issues:

1) Robustness

Even with using such a phenomenological model, the existence of chimera is possible only for identical oscillators, making the usefulness of the model for real systems very limited. Of course, the number of parameters to control for is large, but the authors should investigate the system with noise to account for biological heterogeneity.

Within the current model framework it appears that b is the critical parameter of the model that controls the multidien periodicity, whereas alpha controls the strength of the periodicity. How general is this? Is this robust to network size? The results would also be strengthened if the authors describe the effect of the size of the system as well as diverse network topologies, at least for a couple of representative examples For example, circadian (even endogenous) rhythms exist in the heart, liver, stomach, gut. What is the minimum system required to produce a 7 day or 45 day multidien rhythm? Is this phenomena typical only for ring networks or also for random, small-world or scale-free networks, which more closely resemble biological connectivity. Is there a simple explanation for this division between the effects of these two model parameters?

The model consistently yields a ~7-day peak. While this aligns with "circaseptan" rhythms, there is a risk of circular reasoning: if the network parameters ($b, \alpha, p$) are tuned to match observed data, is the 7-day emergence truly "spontaneous," or is it a byproduct of the specific ring-coupling architecture chosen?

A thorough analysis may be for another study, but providing such data here is important, particularly to show that the phenomenon is not cherry picked or an epiphenomenon of the network choices made.

2) The introduction of the driver which is coupled as a difference.

Driven Kuramoto oscillators are known and studied, also in the context of the circadian rhythms, but the coupling always needs to be through the sine term. There cannot be a derivation of phase oscillators where the coupling enters like in the Eq. 2. (Even if it is true that for small x sin(x) \approx x, this would need to be justified).

3) Line 30: The authors state that an independent multidien clock that synchronizes physiological systems over days seems unlikely. What is the support for this assertion? Clearly there are interacting hormonal cycles that exist over these cycles. Is it the authors' hypothesis that these cycles only emerge from the interactions of circadian oscillators?

4) Line 64: The authors state they do want a synchronous state with 0<R<1.They should clarify what they mean by synchronization or partial synchronization (checking the Synchronization book by Pikovsky should be helpful).

5) The authors found that a 24-hour external cue (like daylight) actually strengthens the multidien cycles. This is counterintuitive and requires deeper validation to ensure it isn't an artifact of the mathematical coupling between the driver and the oscillators or is it again a more robust finding.

Minor:

1) How were the initial phase conditions set for the model?

2) Line 172: The authors state that their network characteristics align with physiological data from Talamanca et al. 2023. In what manner are they aligned? How are they different?

3) The authors test the impact of a circadian zeitgeber on the network and show that it only increases the multidien cycle strength. It is often the case that the zeitgeber changes - for example by a change in time zone. Do such shifts in zeitberger lead to systematic multidien cycle changes?

4) There are a few typos, such as instances of Kumamoto written with small letter

5) Fig. 2b, what is z? should it be R(t)?

## Author Response

--------------------------------------------- Synthesis of Reviews:

Computational Neuroscience Model Code Accessibility Comments for Author (Required):

Please include a section title Code Accessibility in the Methods section indicating how the code can be accessed.

We added a subsection in the methods that shows how to get the codes to reproduce the results of this manuscript.

Code Accessibility All scripts used to reproduce the results presented in this manuscript are publicly available at [URL redacted for double-blind review].

Synthesis Statement for Author (Required):

Two reviewers evaluated the manuscript "Emergent multidien cycles from partial circadian synchrony." The authors describe a theory for how long cycles, with periods extending from weeks to months, could emerge from a circadian rhythm. Both reviewers agreed that the manuscript is thought-provoking and provides a compelling account for a clinically relevant issue. The reviewers raised a few issues which are detailed below. Addressing these issues should strengthen the rigor of the manuscript and clarify the analyses and interpretations of the work.

We thank the Editor and the Reviewers for their thorough assessment of the manuscript. We believe that the suggested analyses and clarifications have substantially strengthened the work, and we hope the revised manuscript is now suitable for publication.

Major Issues:

1) Robustness Even with using such a phenomenological model, the existence of chimera is possible only for identical oscillators, making the usefulness of the model for real systems very limited. Of course, the number of parameters to control for is large, but the authors should investigate the system with noise to account for biological heterogeneity.

Regarding the use of identical oscillators, our assumption is well-founded on the established basis that virtually every cell in the body harbours an autonomous molecular clock, driven by the transcription-translation feedback loop (TTFL) in which the CLOCK:BMAL1 heterodimer activates the transcription of the Period (Per1, Per2) and Cryptochrome (Cry1, Cry2) genes, whose protein products in turn translocate to the nucleus and repress CLOCK:BMAL1 activity, completing the ~24 h cycle [1,2]. Our hypothesis is not that all oscillators are identical, but rather that they share the same core clock architecture and intrinsic period properties.

To address this concern explicitly, we tested our model under conditions of biological heterogeneity by introducing variability in the natural frequency of the oscillators and added Fig. 3-2. Our results show that the emergent periodicity is preserved under moderate heterogeneity; however, as the noise increases, the power at the 24 h period becomes progressively more dispersed (Fig. 3-2a,b). Here is the modified text in the methods:

Finally, we introduced variability in the intrinsic frequency of each oscillator by perturbing the baseline natural frequency with multiplicative Gaussian noise. In practice, for a baseline frequency ⍵, we defined the oscillator-specific frequencies as where σ controls the amount of heterogeneity, so the frequencies have a normal distribution centered at 1 with standard deviation σ. This means that the natural frequencies are no longer identical, but are spread around the reference value ⍵. Unless stated otherwise, we keep the variability at zero.

And in the result text:

Multidien periodicities also arise when all oscillators share a natural frequency close to the circadian value, over a range of 20-28 h (Fig.3-1), showing that the effect is not specific to a single 24 h natural period. Heterogeneity in the natural frequencies of the oscillators contrary disrupts periodicity, with increased frequency dispersion progressively degrading the regularity of multidien cycles (Fig.3-2). This suggests that heterogeneity in the natural frequencies can support multidien cycles over a finite range, but when it becomes sufficiently large, it disrupts the collective dynamics and destroys the quasi-periodic pattern.

Fig.3-2. Strong heterogeneity destroys the emergence of multidien rhythms. a) Time evolution of the order parameter for different levels of natural-frequency heterogeneity. b) Periodogram of the model with different levels of noise strength σ using b = 15 and α = 1.48 Within the current model framework it appears that b is the critical parameter of the model that controls the multidien periodicity, whereas alpha controls the strength of the periodicity. How general is this? Is this robust to network size? The results would also be strengthened if the authors describe the effect of the size of the system as well as diverse network topologies, at least for a couple of representative examples For example, circadian (even endogenous) rhythms exist in the heart, liver, stomach, gut. What is the minimum system required to produce a 7 day or 45 day multidien rhythm? Is this phenomena typical only for ring networks or also for random, small-world or scale-free networks, which more closely resemble biological connectivity. Is there a simple explanation for this division between the effects of these two model parameters? We agree with the Reviewers that a more comprehensive study of the robustness of our model across different sizes and networks was needed.

Regarding network size, we added a new supplementary figure (Fig. 3-3) showing that the system is robust across different sizes. When the system size is small, periodicity becomes slightly unstable, but stability improves consistently as the network grows.

We also investigated the effect of different network topologies. In the original manuscript, we examined what happens when links are randomly removed with an increasing probability p, yielding results for networks increasingly similar to a random network, as shown in Fig. 3c-d. However, we recognise that a broader demonstration across other topologies strengthens the analysis. The ring topology with nonlocal interactions is the canonical and most widely studied setting for chimera states, which is why the manuscript focuses on it. To complement this, we incorporated a Barabási-Albert (BA) network into the model, with results shown in Fig. 6. Multidien rhythms can indeed be recovered in this topology, confirming that the findings reported in the manuscript are not exclusive to ring networks. A full parametric study of the BA topology would, however, require a more extensive analysis: not only does the phase lag interact differently with the scale-free structure, but for the same construction parameter, multiple network realisations are equally possible depending on the seed of the preferential attachment algorithm.

Finally, regarding the parameter roles, b governs the overall network topology, which is why it has a stronger influence on global periodicities. The phase lag, by contrast, modulates the interactions between individual oscillators - when low enough, it drives the entire network towards synchronisation.

All the new analysis and modified text are here in red:

These effects were consistently found across networks of different sizes (Fig.3-3) Fig.3-3. Periodogram of the order parameter R(t) for network sizes N⋹ (20, 50, 100, 200, 500). Line color progresses from light to dark red with increasing N, with coupling parameter b~0.3N (scaled such that b = 15 at N = 50) and ⍺= 1.48 fixed throughout. A dominant peak around ~35 days is consistently recovered across all system sizes, confirming the robustness of the oscillatory dynamics. The broadening observed for N = 20 reflects reduced network stability at small sizes.

Throughout the manuscript, we explore ring network topologies, as they represent the canonical setting in which chimera states and partial synchronisation have been studied most extensively. However, biological systems such as protein-protein interaction networks are known to exhibit scale-free topology [3], characterised by a few highly connected hubs and many weakly connected nodes. To account for this, we simulated a network generated via the Barabási-Albert preferential attachment algorithm [4] (Fig.6b): starting from m0 initial nodes, at each step a new node is added and connects to m existing nodes with a probability proportional to their degree - the so-called "rich-get-richer" mechanism that gives rise to the scale-free degree distribution. Although a full exploration of the parameter space would require a more extensive study, we found a parameter set for which the system does not reach full synchronisation (0 < R(t) < 1), as shown in Fig. 6a. Under these conditions, significant multidien periodicities (Fig 6c) in R(t) can indeed be recovered, demonstrating that the emergence of multidien rhythms is not exclusive to ring topologies.

Fig.6. Multidien cycles are found in scale-free networks. a) Order parameter across time of a window of the simulation. b) Barabási-Albert network example for m0=6, and m=2.c) Periodogram of the order parameter for the system simulated with the network in b) and with ⍺= 0.6.

The model consistently yields a ~7-day peak. While this aligns with "circaseptan" rhythms, there is a risk of circular reasoning: if the network parameters ($b, \alpha, p$) are tuned to match observed data, is the 7-day emergence truly "spontaneous," or is it a byproduct of the specific ring-coupling architecture chosen? This is a relevant concern. The parameters in our model are chosen based on their ability to generate chimera-like dynamics - that is, the synchronisation of a subset of oscillators while the remaining ones stay asynchronous. For a large portion of the parameter space, this behaviour does not emerge, and therefore, parameter selection is driven by the requirement to generate a chimera state. Of note, we do not tune any parameter to achieve a desired periodicity. Instead, the region of parameter space in which chimera-like behaviour exists naturally coincides with systems that tend to exhibit circaseptan and approximately monthly periodicities, provided that the natural frequency is one day.

A thorough analysis may be for another study, but providing such data here is important, particularly to show that the phenomenon is not cherry picked or an epiphenomenon of the network choices made.

The concern is pertinent. We have added multiple new analyses covering different network sizes, topologies, and driver periodicities, which together demonstrate that the observed phenomenon is not cherry-picked nor an artefact of the specific network choices made.

2) The introduction of the driver which is coupled as a difference.

Driven Kuramoto oscillators are known and studied, also in the context of the circadian rhythms, but the coupling always needs to be through the sine term. There cannot be a derivation of phase oscillators where the coupling enters like in the Eq.

2. (Even if it is true that for small x sin(x) \approx x, this would need to be justified).

The reviewers are correct. We apologise for the error - the equation as written in the manuscript was incorrect. The code indeed uses a sine function for the interaction between the driver phase and the phase of oscillator i. This has been corrected in the revised manuscript.

3) Line 30: The authors state that an independent multidien clock that synchronizes physiological systems over days seems unlikely. What is the support for this assertion? Clearly there are interacting hormonal cycles that exist over these cycles. Is it the authors' hypothesis that these cycles only emerge from the interactions of circadian oscillators? We acknowledge that this remains a hypothesis. The diversity of multidien periods observed across physiological systems, together with their quasi-rhythmic nature, is difficult to reconcile with a rigidly hard-coded biological clock. We therefore propose, as one possible explanation, that chimera-like network dynamics may underlie the generation of these flexible, system-level periodicities. An alternative hypothesis, however, is that hormones - whose own multidien rhythmicity exists but whose governing clock remains unidentified - act as synchronising signals across physiological systems throughout the body. Distinguishing between these two mechanisms represents an important open question for future work.

4) Line 64: The authors state they do want a synchronous state with 0<R<1.They should clarify what they mean by synchronization or partial synchronization (checking the Synchronization book by Pikovsky should be helpful).

We thank the reviewer for this suggestion. In this manuscript, synchronisation refers to the state in which all oscillators share the same phase, corresponding to an order parameter R = 1 in the Kuramoto framework. As we state, this is precisely the state we aim to avoid. Instead, our model operates in the regime of partial synchronisation, specifically in the form of chimera states, where a subset of oscillators locks into coherent, synchronised dynamics while the remaining oscillators behave incoherently and asynchronously - yielding an order parameter 0 < R < 1. This distinction is carefully discussed in the seminal Synchronization book by Pikovsky, Rosenblum and Kurths, and chimera states themselves have been extensively studied within the same theoretical community, including contributions from Professor Pikovsky on partial synchronisation phenomena in coupled oscillator networks. We have clarified this terminology in the revised manuscript.

As we want to evaluate how R(t) varies over time, we are only interested in configurations that do not lead to a synchronous state (0<R(t)<1) and exclude the realizations that collapse to the fully synchronous state (R(t)=1) within the simulation time.

5) The authors found that a 24-hour external cue (like daylight) actually strengthens the multidien cycles. This is counterintuitive and requires deeper validation to ensure it isn't an artifact of the mathematical coupling between the driver and the oscillators or is it again a more robust finding.

We agree that these findings merit further validation. To address this, we have added a new panel in Fig. 5, in which we examine the effect of different external cues on the model. Interestingly, varying the cue does not substantially alter the original period - in the case shown, the approximately monthly rhythm remains stable. However, the closer the driver frequency is to 24 hours, the stronger the circaseptan rhythm emerges, as reflected by its higher spectral power. This demonstrates that the alignment between the external driver and the natural frequency of the system is a key condition for this behaviour to arise. Here is the modified text and modified main figure:

To assess the importance of frequency-matched pacing, we varied the Zeitgeber's period and examined its effect on the magnitude of emergent periodicities. We observed that about-weekly periodicity is strengthened only when the Zeitgeber frequency is close to the natural frequency of the system (here ~24h). Even a 7-day (i.e. calendar week) did not strengthen the magnitude of endogenously-generated multidien cycles. This shows that a Zeitgeber specifically set at around 24 hours can enhance self-organized multidien cycles of about a week, while leaving about-monthly periodicity unaffected.

Fig.5. The addition of a circadian Zeitgeber in the system of oscillators enhances the magnitude of multidien cycles. a) Partial synchronization example with an arrow showing when the Zeitgeber is added to the system of oscillators (ε=0.1). The lower plot depicts the overall power between 4-10 days of the order parameter oscillations. b) Periodogram of the order parameter as a function of Zeitgeber strength ε, keeping b=15, and ⍺=1.42. c) Change of the power in the multidien range keeping ⍺ = 1.50 and varying the coupling width b and the Zeitgeber strength ε. d) Periodogram of the order parameter as a function of the driver period, keeping ε=0.1, ⍺=1.42, and b=15. Thick lines correspond to driver-period longer than 24h and dotted lines to driver-period shorter than 24h, with redder colors the bigger the separation from 24h.

Minor:

1) How were the initial phase conditions set for the model? We set the initial conditions to a random uniform distribution between 0 and 2 pi. We added the information in the method section.

The initial conditions are drawn from a random uniform distribution between 0 and 2ⲡ.

2) Line 172: The authors state that their network characteristics align with physiological data from Talamanca et al. 2023. In what manner are they aligned? How are they different? In that study, the Talamanca et al. use gene expression data from the Genotype-Tissue Expression (GTEx) project and apply an algorithm to estimate the circadian phase of each tissue in individual donors. Interestingly, they find that phases are broadly aligned across tissues but not fully synchronised - the majority of tissues share similar phases, but not all of them. This pattern closely mirrors the behaviour we report: most circadian oscillators in our model are synchronised, while the remainder exhibit a spread of distinct phases. We have rephrased the corresponding sentence in the manuscript to improve clarity.

Furthermore, key network characteristics - such as the Kuramoto order parameter - align with those found in circadian gene expression across 46 human tissues, where phases cluster around a common mean while a minority of tissues display divergent phases, reminiscent of the partial synchronization of chimera states.

3) The authors test the impact of a circadian zeitgeber on the network and show that it only increases the multidien cycle strength. It is often the case that the zeitgeber changes - for example by a change in time zone. Do such shifts in zeitberger lead to systematic multidien cycle changes? This is an interesting question. In Fig. 5-1, we show that when the driver shifts its phase - analogous to seasonal changes in the light-dark cycle - the network adjusts its phase accordingly. A more systematic investigation of the effects of zeitgeber disruptions such as jet lag or time zone shifts goes beyond the scope of the present manuscript, but represents an interesting avenue for future work.

4) There are a few typos, such as instances of Kumamoto written with small letter Thanks for pointing them out. We corrected them.

5) Fig. 2b, what is z? should it be R(t)? The Reviewer is correct. We have amended the notation and carefully checked the manuscript to ensure no further inconsistencies remain.

References [1] Reppert, S.M. &Weaver, D.R. Coordination of circadian timing in mammals.

Nature 418, 935-941 (2002). [2] Takahashi, J.S. Transcriptional architecture of the mammalian circadian clock.

Nature Reviews Genetics 18, 164-179 (2017). [3] Wuchty, S. (2001). Scale-free behavior in protein domain networks. Molecular biology and evolution, 18(9), 1694-1702. [4] A.-L. Barabasi and R. Albert, "Emergence of scaling in random networks," science, vol. 286, no. 5439, pp. 509-512, 1999.
