# Supplementary figures and images for: Emergent Multidien Cycles From Partial Circadian Synchrony
Source: eNeuro. 2026 Jul 15;13(7):ENEURO.0464-25.2026. doi: 10.1523/ENEURO.0464-25.2026 (PMC13379366; doi:10.1523/ENEURO.0464-25.2026)

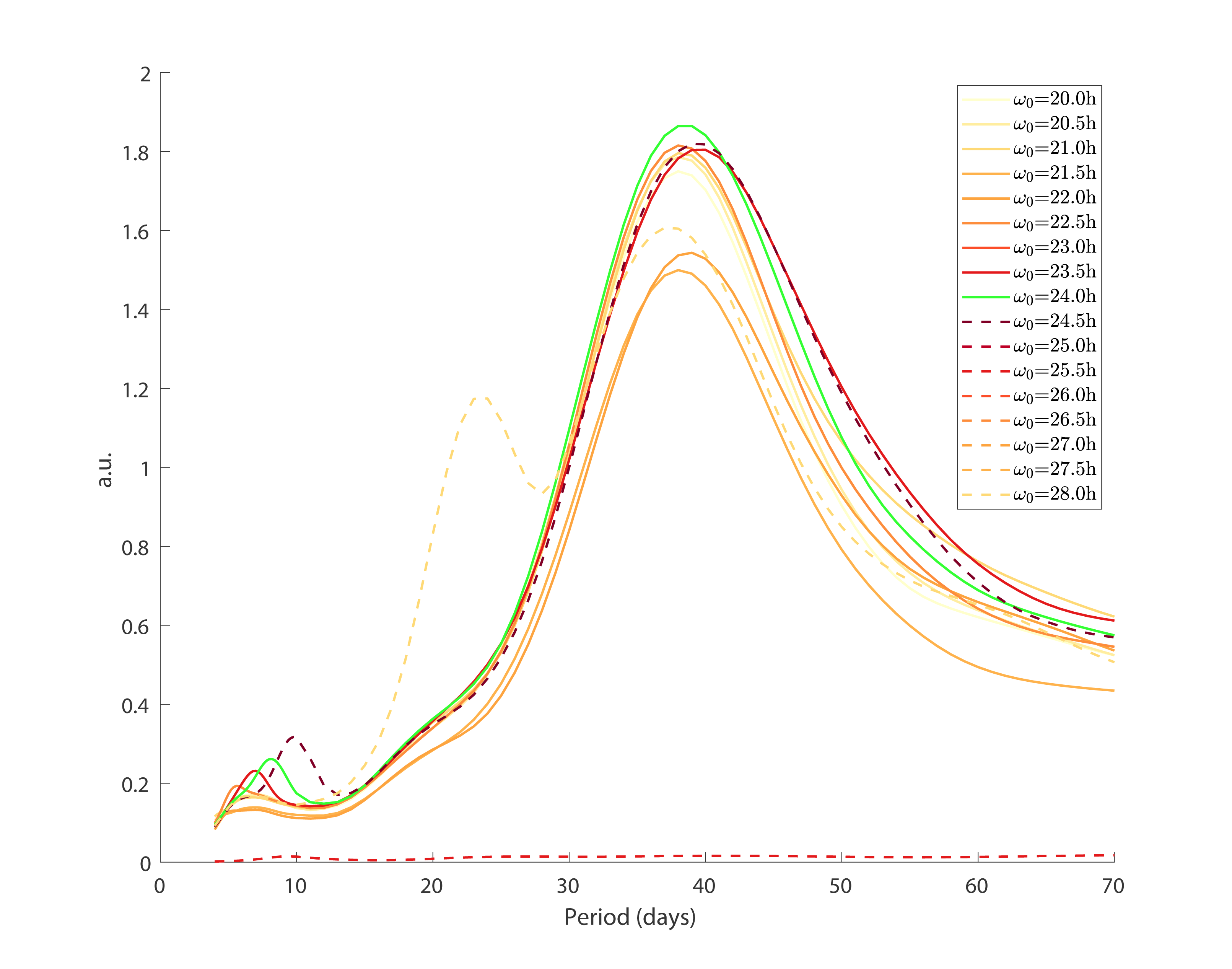

Supplement: Figure 3-1 — Changing the natural frequency around the 24 h period also generates multidien periodicities a) Periodogram of the model with driving using ɛ = 0.05, b = 15 and α = 1.42 as a function of the natural frequency ω0. The green line depicts our original setup with the natural frequency equal to the driver frequency. Solid (dashed) depicts periods lower (higher) than the natural frequency. Download Figure 3-1, TIF file. [file eneuro-13-ENEURO.0464-25.2026-s002.tif]

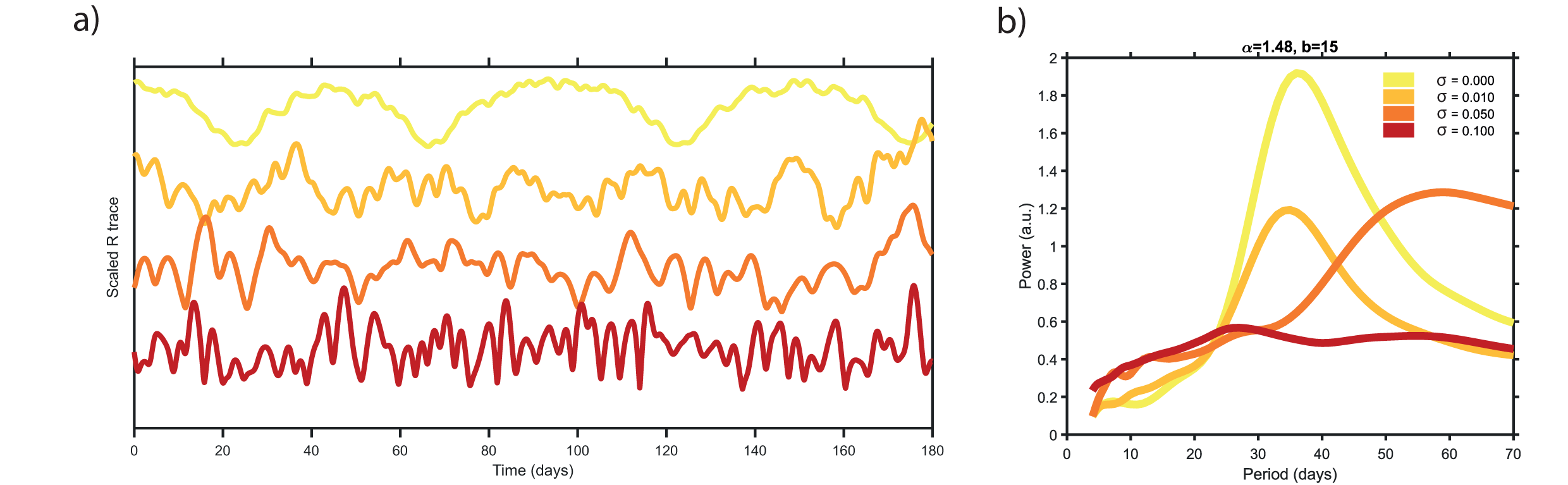

Supplement: Figure 3-2 — Strong heterogeneity destroys the emergence of multidien rhythms a) Time evolution of the order parameter for different levels of natural-frequency heterogeneity. b) Periodogram of the model with different levels of noise strength σusing b = 15 and α = 1.48 Download Figure 3-2, TIF file. [file eneuro-13-ENEURO.0464-25.2026-s003.tif]

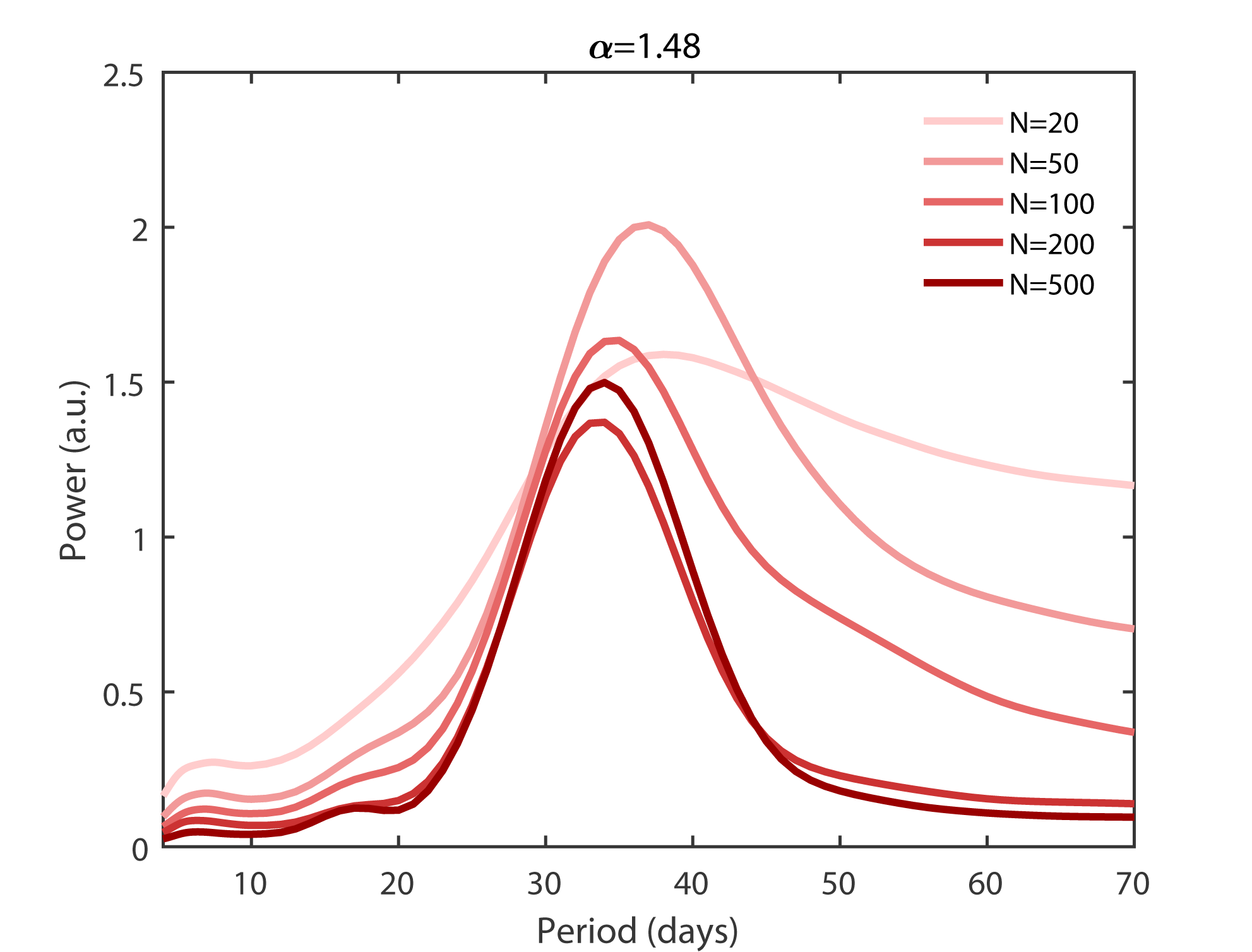

Supplement: Figure 3-3 — Size of the network does not affect the multidien rhythms Periodogram of the order parameter R(t) for network sizes N ∈ {20, 50, 100, 200, 500}. Line color progresses from light to dark red with increasing N, with coupling parameter b = 0.3N (scaled such that b = 15 at N = 50) and α = 1.48 fixed throughout. A dominant peak around 35 days is consistently recovered across all system sizes, confirming the robustness of the oscillatory dynamics. The modest broadening observed for N = 20 reflects reduced network stability at small sizes. Download Figure 3-3, TIF file. [file eneuro-13-ENEURO.0464-25.2026-s004.tif]

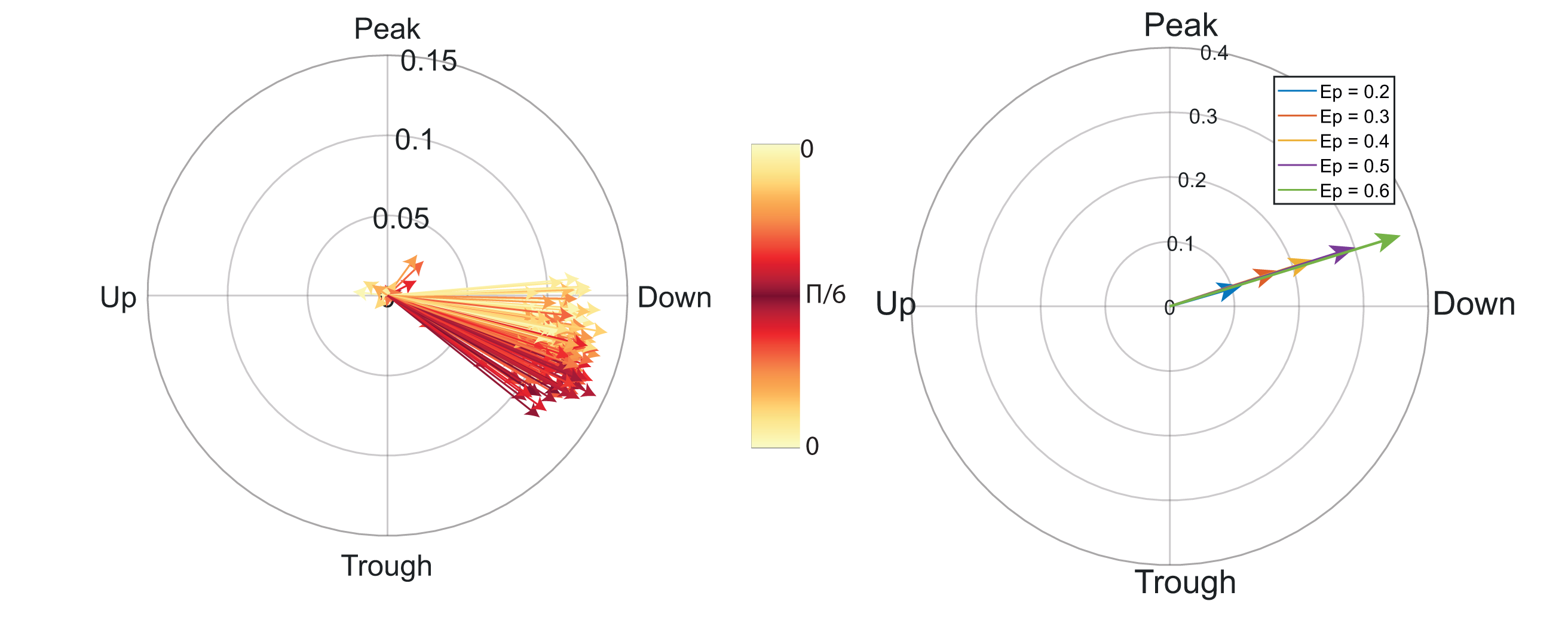

Supplement: Figure 5-1 — The IEA locking with the sun has a similar effect as the change of locking of I(t) with the time as αD(t) changes. a) Locking between the simulated IEA and the time of the day while changing αD between 0 and π/6 in one year of simulation. Each arrow represents a monthly average locking, which corresponds to 600 data points. b) Locking between the IEA and the time of the day for different ǫ keeping αD = −π/6. Download Figure 5-1, TIF file. [file eneuro-13-ENEURO.0464-25.2026-s005.tif]
